# Evidence Showing that Tetraspanins Inhibit HIV-1-Induced Cell-Cell Fusion at a Post-Hemifusion Stage

**DOI:** 10.3390/v6031078

**Published:** 2014-03-07

**Authors:** Menelaos Symeonides, Marie Lambelé, Nathan H. Roy, Markus Thali

**Affiliations:** 1 Graduate Program in Cell and Molecular Biology, University of Vermont, Burlington, VT 05405, USA; E-Mails: msymeoni@uvm.edu (M.S.); nroy@uvm.edu (N.H.R.); 2 Department of Microbiology and Molecular Genetics, University of Vermont, Burlington, VT 05405, USA; E-Mail: mlambele@uvm.edu

**Keywords:** tetraspanin, CD9, CD63, HIV, Env, cell-cell fusion, hemifusion

## Abstract

Human immunodeficiency virus type 1 (HIV-1) transmission takes place primarily through cell-cell contacts known as virological synapses. Formation of these transient adhesions between infected and uninfected cells can lead to transmission of viral particles followed by separation of the cells. Alternatively, the cells can fuse, thus forming a syncytium. Tetraspanins, small scaffolding proteins that are enriched in HIV-1 virions and actively recruited to viral assembly sites, have been found to negatively regulate HIV-1 Env-induced cell-cell fusion. How these transmembrane proteins inhibit membrane fusion, however, is currently not known. As a first step towards elucidating the mechanism of fusion repression by tetraspanins, e.g., CD9 and CD63, we sought to identify the stage of the fusion process during which they operate. Using a chemical epistasis approach, four fusion inhibitors were employed in tandem with CD9 overexpression. Cells overexpressing CD9 were found to be sensitized to inhibitors targeting the pre-hairpin and hemifusion intermediates, while they were desensitized to an inhibitor of the pore expansion stage. Together with the results of a microscopy-based dye transfer assay, which revealed CD9- and CD63-induced hemifusion arrest, our investigations strongly suggest that tetraspanins block HIV-1-induced cell-cell fusion at the transition from hemifusion to pore opening.

## 1. Introduction

HIV-1 enters target cells by fusing the viral lipid envelope with the cell membrane, allowing the release of the viral core into the cell (reviewed in [1]). The viral envelope glycoprotein Env, however, not only mediates fusion of viral and cellular membranes, but can also drive the fusion of the plasma membranes of infected and uninfected cells, thus giving rise to the formation of multinucleated entities, *i.e.*, syncytia. HIV-1-induced syncytia are readily observable not only in cell culture systems but have also been documented in infected individuals [2,3,4] as well as in a humanized mouse model [5], though a quantification of how many infected cells exist as syncytia *in vivo* is not feasible. However, *in vitro* analyses as well as certain *in vivo* observations [6,7] suggest that, in the majority of cases, contacts between infected and uninfected cells, which can lead to particle transmission via the virological synapse ([8], and for a recent review, see [9]), dissolve without resulting in cell-cell fusion.

While, theoretically, virus dissemination through a succession of syncytia is possible because syncytia produce large amounts of progeny viruses [10], it has been established that syncytia tend to undergo apoptosis (reviewed in [11,12]). Also, establishment of latency (for a review, see [13]) is likely not possible in these short-lived syncytia. Thus, fusion regulation, beyond simply controlling proper timing of the viral entry process, may have evolved to ensure continued virus spread through particle transmission without cell-cell fusion. Indeed, by now, several ways by which HIV-1 regulates the fusogenicity of Env have been identified. These include: (a) the rapid internalization of newly synthesized Env from the surface of the infected cell (reviewed in [14]); (b) an interaction between the cytoplasmic tail of the gp41 transmembrane domain of Env and the matrix domain of immature Gag, which strongly represses the fusogenicity of Env not only within the virion, but also already at the virological presynapse [15,16,17,18,19]; and (c) the active recruitment of tetraspanins to viral assembly sites [20,21], where they repress cell-cell fusion [22] and, upon their acquisition by newly formed particles, virus-cell fusion [23,24]. An involvement of tetraspanins in the regulation of Env-induced membrane fusion should not be surprising, as these proteins have been shown to regulate numerous membrane fusion processes, including mammalian spermatocyte-oocyte fusion (reviewed in [25]), macrophage fusion [26,27], and myoblast fusion [28,29]. Indeed, a very recent report also implicates a tetraspanin in yet another virus-triggered membrane fusion event [30].

**Figure 1:**
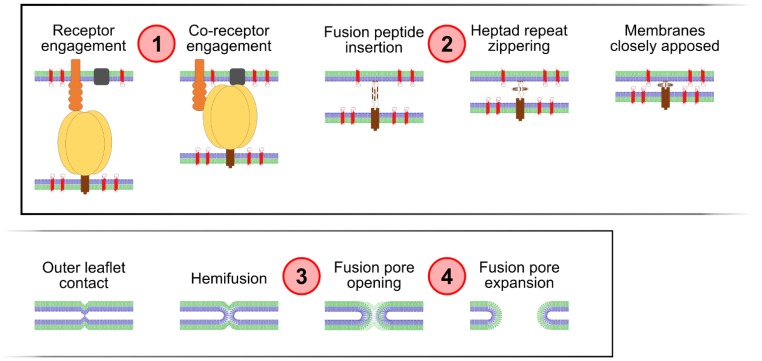
Schematic of fusion steps, and stage of action of fusion inhibitors used, denoted as numbered red circles: (**1**) AMD3100; (**2**) C34; (**3**) Imatinib; and (**4**) dynasore. Schematic key: Outer membrane leaflets–blue; Inner membrane leaflets–green; HIV-1 Env gp120–yellow, gp41–brown (cytoplasmic tail not shown); CD4–orange; CXCR4–grey; Tetraspanins–red.

How tetraspanins regulate membrane fusion, in any context, is currently unknown. In order to pave the way towards understanding the mechanism of fusion regulation by these proteins as well as the involvement of potential cofactors, we sought to determine which stage of Env-induced fusion is affected by tetraspanins (see [31,32] for detailed descriptions of the HIV-1 Env-induced fusion reaction, and Figure 1 for a schematic of the steps involved). To achieve this, we monitored Env-induced cell-cell fusion while applying a panel of fusion inhibitors that operate at different stages of fusion in tandem with tetraspanin overexpression (*i.e.*, a chemical epistasis approach), as well as by using an imaging-based fluorescent dye transfer assay. 

## 2. Results and Discussion

### 2.1. Temporal Delineation of CD9-Mediated Fusion Repression Using Chemical Epistasis

Previously, we and others reported that overexpression of the tetraspanins CD9 and CD63 (as well as other members of this family of membrane scaffolds) in HIV-1-producing cells represses virus-cell and cell-cell fusion induced by Env [22,23,24]. In contrast, L6, a host transmembrane tetraspanin-like protein (also known as TM4SF1) has no such effect, despite the fact that it colocalizes with tetraspanins at the plasma membrane and is incorporated into HIV-1 particles [23,24,33]. Applying a chemical epistasis approach in order to identify the step in the fusion process affected by CD9 overexpression, we used four fusion inhibitors with known action (the steps of the fusion process they affected are delineated in Figure 1). The four inhibitors target: (1) the co-receptor engagement stage (the small molecule AMD3100, which blocks CXCR4); (2) the prehairpin intermediate (the C-peptide C34); (3) the hemifusion intermediate (the Abl kinase inhibitor Imatinib [34]); and (4) pore expansion (the small molecule dynamin inhibitor dynasore [34,35,36]). It should be noted that the fusion inhibitory action of Imatinib has only been reported once so far, and the underlying mechanism is not yet clear. Similarly, that dynasore affects pore expansion is a relatively recent finding. Consequently, the action of both inhibitors will thus likely be the subject of future scrutiny. Given, however, the lack of more established inhibitors of these stages of the fusion process, we decided nevertheless to use them here. Inhibitor concentrations were empirically determined in order to span a range of fusion inhibition (data not shown) and five different concentrations of inhibitor in addition to a vehicle control were employed in each case.

**Figure 2:**
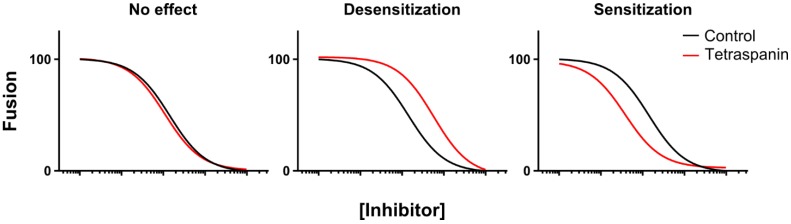
Chemical epistasis approach; possible outcomes of tandem tetraspanin overexpression with fusion inhibitor titration.

**Figure 3:**
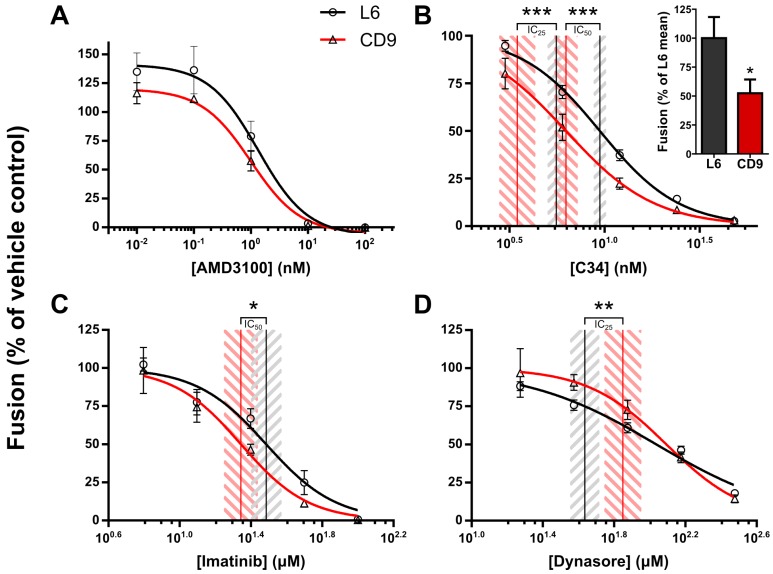
Chemical epistasis experiments using fusion inhibitors AMD3100 (**A**); C34 (**B**); Imatinib (**C**); and dynasore (**D**) in virus-producing HeLa cells overexpressing L6 or CD9 co-cultured with TZM-bl target cells. Fusion levels were quantified using the DSP fusion assay. The basal effect of L6 and CD9 overexpression on fusion (shown here as an inset in **B**; data taken from the same experiment) was equalized by normalizing to vehicle. The data points were fitted to a classical inhibition response model and the inhibitor concentrations leading to 25% inhibition (IC_25_) or 50% inhibition (IC_50_) were compared between L6 and CD9 for each inhibitor. Shown are the only comparisons which exhibited a significant difference.

Cell-cell fusion assays were carried out using the dual split protein (DSP) system developed by the Matsuda group [37,38], whereby fusion can be measured by the development of GFP fluorescence in syncytia upon bimolecular complementation. HeLa cells were co-transfected with HIV-1 pNL4-3 and L6 or CD9, and later co-cultured with TZM-bl cells, while fusion inhibitors were titrated in so that an inhibition profile could be obtained by fitting an inhibition model (as described in the Experimental section; Figure 2). The basal effect of CD9 overexpression on the level of fusion (typically a ~50% reduction in this assay, as shown in Figure 3B, inset) was eliminated by normalizing to the level of fusion when vehicle alone was used. The inhibitory concentrations leading to 25% or 50% inhibition (IC_25_ or IC_50_) for each inhibitor could then be compared between the L6 and CD9 overexpression treatment. Possible outcomes of such experiments are depicted in Figure 2 and described in the following. If there was no difference in IC_25_ or IC_50_ between L6 and CD9, we would conclude that the inhibitor acted before CD9, or that the inhibitor was epistatic over CD9 (Figure 2, left panel). However, if the CD9 treatment exhibited a desensitization to the inhibitor (an increased IC_25_ or IC_50_), we would conclude that the effect of CD9 was epistatic over the inhibitor (Figure 2, middle panel). Furthermore, if sensitization was observed (a reduced IC_25_ or IC_50_), we interpreted that as the inhibitor again being epistatic over CD9, but the action of CD9 is in some way affecting the inhibitor’s action (Figure 2, right panel).

The results of our analyses are shown in Figure 3. The inhibition profile of AMD3100 within the L6 and CD9 treatments was not significantly different (Figure 3A), and we can thus conclude that CD9 regulates a stage that follows co-receptor engagement. Interestingly, CD9 overexpression led to significant sensitization to the C-peptide C34, indicating that a process which closely follows the formation of the pre-hairpin intermediate was affected by CD9 overexpression (Figure 3B). Similarly, significant sensitization to the Abl kinase inhibitor Imatinib, which has been shown to arrest fusion at the hemifusion intermediate [34], was observed upon CD9 overexpression (Figure 3C), strongly suggesting that CD9 inhibits the fusion process after the hemifusion intermediate has formed. Furthermore, and strikingly, when the dynamin inhibitor dynasore (which impedes pore expansion [36]) was titrated, CD9 overexpression led to significant desensitization to this inhibitor (Figure 3D), showing epistasis of CD9 over dynasore in fusion repression. Together, these results allowed us to conclude that CD9 regulates a stage of fusion concurrently with or after lipid mixing and prior to pore expansion. 

### 2.2. Tetraspanin Overexpression Leads to Accumulation of the Hemifusion Intermediate

**Figure 4:**
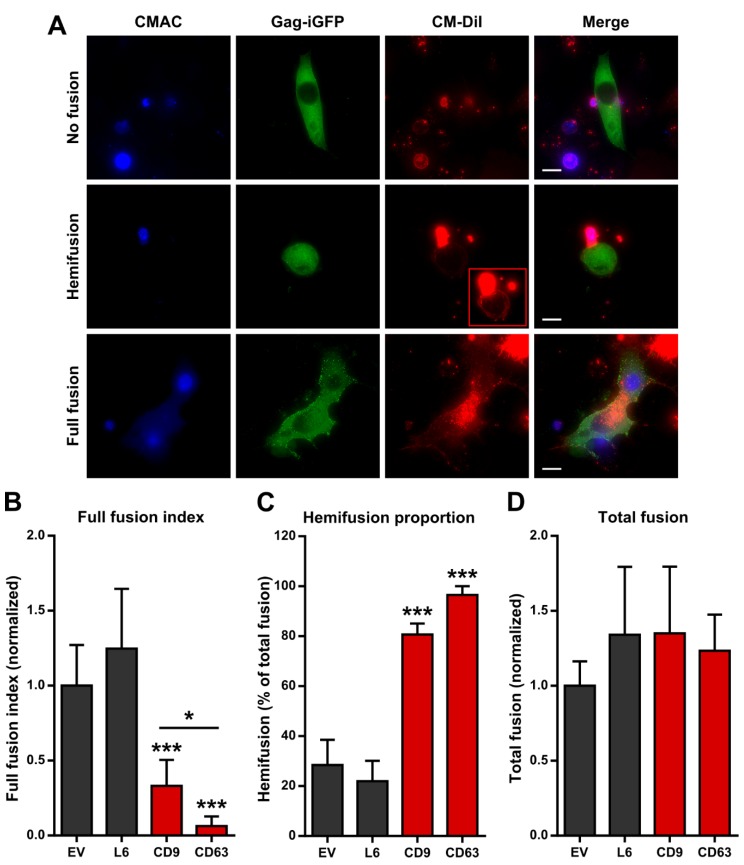
Accumulation of the hemifusion intermediate caused by tetraspanin overexpression. (**A**) HeLa cells transfected with pNL4-3^Gag−iGFP^ and the indicated plasmids were co-cultured with CMAC and CM-DiI-labeled Jurkat cells, fixed, and imaged. The three possible outcomes of cell-cell contact are shown as examples. Inset in Hemifusion/CM-DiI panel is contrast-enhanced. Bar = 10 μm; (**B**) Quantification of full fusion. The number of CMAC-positive nuclei found within GFP-positive syncytia was divided by the number of GFP-positive cells and normalized to the empty vector control; (**C**) Quantification of hemifusion (cells positive for GFP and CM-DiI, but not CMAC) as a percentage of the sum of hemifusions and syncytia; (**D**) Total fusion activity measured as the sum of hemifusions and syncytia, normalized to the empty vector control. Statistical comparisons made against L6, except where denoted by a horizontal bar.

Based on the results shown in Figure 3, which indicate that tetraspanins act at a post-hemifusion stage, we sought to further test this hypothesis using a different, more direct approach. We employed an imaging-based fluorescent dye transfer assay [39,40], whereby target Jurkat T cells labeled with the cytosolic dye CMAC and the fixable lipophilic dye Vybrant CM-DiI were co-cultured with producer HeLa cells transfected with pNL4-3^Gag−iGFP^ and a tetraspanin or the L6 control. After 5 h of co-culture, excess unbound target cells were washed off, and the cultures were imaged after fixation. Gag-iGFP-positive cells were counted, and the proportion of those which were positive for CM-DiI alone was taken as indicative of reactions that had reached the hemifusion stage, while the proportion which was positive for both CM-DiI and CMAC (visible as an additional nucleus in the syncytium and corresponding to a gap in the GFP channel) was taken as quantification of full fusion or syncytium formation. Example images of each of these cases are shown in Figure 4A.

As expected, reduced levels of full fusion (measured as the number of CMAC-positive nuclei in infected syncytia divided by the number of infected cells) were consistently observed when CD9 or CD63 were overexpressed compared to the L6 or empty vector controls (Figure 4B). However, taking into account total fusion activity (hemifusions plus syncytia), we found a striking increase in the hemifusion proportion upon tetraspanin overexpression (Figure 4C). This indicates that tetraspanin overexpression allowed fusing cells to progress to the hemifusion intermediate, but arrested the process at a later stage, leading to a build-up of this intermediate. Notably, the total fusion activity upon CD9 or CD63 overexpression was no different than that in the L6 control (Figure 4D), meaning that the reduction in full fusion could be fully recovered at the hemifusion stage. This indicates that there is likely no pre-hemifusion repression effect of tetraspanin overexpression.

Taken together, the results of these experiments render strong support for the hypothesis that CD9 and CD63 regulate HIV-1-induced cell-cell fusion after the outer membrane leaflets of the fusing cells have merged and prior to pore expansion. Considering potential mechanisms, we can envision two scenarios: tetraspanins may interact directly with viral or host factors which regulate pore stabilization and/or expansion; alternatively (or additionally), tetraspanins may modulate the mechanical properties of membranes in a way that renders fusion pore stabilization and/or expansion less energetically favorable. The former scenario may involve, for example, cytoskeletal and associated proteins (in unpublished data we find that depletion of ezrin in HIV-1 producing cells leads to enhanced Env-induced cell-cell fusion [41]). Support for the latter possibility comes from studies on the effect of virion stiffness on virus-cell fusion [42,43]; tetraspanins at sites of fusion may similarly increase the local stiffness of membrane (we are currently testing this hypothesis). The sensitization to C34 observed in our experiments (Figure 3B) would also be in line with this potential mechanism; that is, the action of C34 comes at a stage where the apposed membranes of the fusing cells are being brought closer using folding energy, which acts against not only the water molecules found in the intercellular space, but also against the stiffness of the apposed membranes. A stiffer membrane would conceivably slow down the zippering of the heptad repeat regions, thus allowing better opportunity for C34 to bind, leading to sensitization.

Irrespective of the precise mechanism of tetraspanin-mediated fusion repression, the hemifusion intermediate is an important facet of HIV-1 pathogenesis. Bystander cell killing, one of the mechanisms thought to contribute to T cell depletion, can be triggered by transient hemifusion between infected and uninfected cells, resulting in apoptotic death of the uninfected cell (an event sometimes referred to as “kiss of death”, first described in [44] and reviewed in [45,46]). Hence, although tetraspanins are actively recruited by the virus [20,21] to prevent excessive syncytium formation and thus likely promoting efficient virus spread, by arresting Env-induced fusion at the hemifusion stage, these proteins may also be co-responsible for one of the key pathogenic features of HIV-1.

## 3. Experimental Section

### 3.1. Cells, Plasmids, Antibodies, and Reagents

The following reagents were obtained through the NIH AIDS Research and Reference Reagent Program, Division of AIDS, NIAID, NIH: TZM-bl cells from Dr. John C. Kappes, Dr. Xiaoyun Wu and Tranzyme Inc, Jurkat clone E6-1 cells from Dr. Arthur Weiss. HeLa cells were obtained from Dr. Eric Cohen. HeLa and TZM-bl cells were maintained in DMEM supplemented with 10% FBS, 100 units/mL penicillin and 100 μg/mL streptomycin. Jurkat T cells were maintained in RPMI 1640 medium supplemented with 10% FBS, 100 units/mL penicillin and 100 μg/mL streptomycin. All experiments were conducted in media supplemented with 10% FBS and without antibiotics.

The following proviral plasmids were used: pNL4-3 and pNL4-3-ΔEnv (KFS) from Dr. Eric Freed (National Cancer Institute, Frederick, MD, USA); pNL4-3^Gag−iGFP^ and pNL4-3-ΔEnv^Gag−iGFP^ from Dr. Benjamin Chen (Mount Sinai School of Medicine, New York, NY, USA). pCMV-Sport6 expression plasmids containing L6, CD9, or CD63 cDNA were provided by Dr. Yoshio Koyanagi (Kyoto University, Kyoto, Japan) and modified with a C-terminal HA tag by standard PCR-based cloning. Expression plasmids Rluc8-DSP^1−7^ and Rluc8-DSP^8−11^ used in the dual split protein assay were provided by Dr. Zene Matsuda (The University of Tokyo, Japan).

### 3.2. Dual Split Protein (DSP)-Based Cell-Cell Fusion Assay

HeLa cells were co-transfected with the HIV-1 pNL4-3 provirus, an overexpression plasmid (L6 or CD9), and a construct encoding DSP-1 (the *N*-terminal half of GFP fused to the *N*-terminal half of Renilla luciferase 8; Rluc8). In parallel, TZM-bl cells, serving as target cells, were transfected with a construct encoding DSP-2 (the C-terminal half of the aforementioned split-Rluc8-GFP protein). 48 h later, the two cell types were co-cultured for 5 h in order for cell-cell fusion to take place. Upon fusion between virus-producing HeLa and target TZM-bl cells and mixing of their contents, the two parts of the DSP assemble through bimolecular complementation and the resulting syncytium develops green fluorescence, as well as the ability to cleave luciferase substrates (not utilized here).

Cell co-cultures were detached using Trypsin-EDTA and fixed using PBS/4% PFA before resuspension in PBS. Flow cytometry was performed on an LSR II cytometer (BD Biosciences) using a 488 nm excitation laser, with 100,000 events recorded per sample. Flow cytometry datasets were analyzed using FlowJo v.X.0.6 software [47]. The proportion of cells which were GFP-positive compared to an Env-deleted control (pNL4-3-ΔEnv) was taken as the rate of cell-cell fusion. This was multiplied with the mean GFP fluorescence intensity within GFP-positive cells (in order to account for multiple fusions within each syncytium) and normalized to the vehicle control within each treatment (L6 or CD9) at 100% fusion. Three to seven biological replicates were performed for each experiment, consisting of two technical replicates at each inhibitor concentration for each treatment. 

### 3.3. Dye Transfer Cell-Cell Fusion Assay

A classical three-color dye transfer cell-cell fusion assay [39,40] was adapted for our purposes. HeLa cells were transfected with a fluorescently tagged HIV-1 provirus (pNL4-3^Gag−iGFP^ or pNL4-3-ΔEnv^Gag−iGFP^) and an overexpression plasmid (empty, L6, CD9, or CD63). 48 h later, transfected HeLa cells were co-cultured with Jurkat cells labeled with CellTracker Blue CMAC and Vybrant CM-DiI (Molecular Probes). After 5 h of co-culture, unattached Jurkat cells were washed off using PBS, and cells were fixed using PBS/4% PFA, before imaging on a DeltaVision deconvolution miscroscope (Applied Precision, Issaquah, WA, USA) using a 10× objective. Randomly selected fields were imaged, and later manually scored for the presence of GFP, CMAC, and CM-DiI within each cell. At least 900 infected cells were scored in each treatment across three biological replicates, each consisting of two technical replicates. Syncytium formation was measured as the proportion of GFP-positive cells which were also positive for CMAC and CM-DiI, normalized to the background rate of fusion in the ΔEnv condition. The full fusion index was measured by dividing the number of CMAC-positive nuclei found within GFP-positive syncytia by the total number of GFP-positive cells scored. Hemifusion was measured as the proportion of GFP-positive cells that also exhibited CM-DiI membrane labeling but no internalized CMAC, background-subtracted using the non-specific CM-DiI transfer observed in the ΔEnv control. Finally, hemifusion was also measured as a proportion of total fusion activity by dividing the number of hemifusions by the sum of hemifusions and syncytia.

### 3.4. Imaging

Fluorescence images shown in Figure 4A were acquired from cells prepared and co-cultured as described in the dye transfer assay, using the 60× objective on a DeltaVision deconvolution microscope (Applied Precision, Issaquah, WA, USA). Images were deconvolved, Z-stacks were projected, contrast and gamma adjustments were made, and the same adjustment was used for all images displayed.

### 3.5. Statistical Analysis

Statistical tests and curve fitting were performed using GraphPad Prism version 6.01 for Windows (GraphPad Software, San Diego, CA, USA). Cell-cell fusion data using the dual split protein assay were analyzed by nonlinear regression using a standard variable slope inhibitor response model, and comparing the IC_50_ and IC_25_ values between conditions using an extra sum-of-squares F test. Three-color dye transfer fusion assays were analyzed by two-way analysis of variance and differences between conditions were evaluated using Fisher’s LSD Test. Correction for multiple comparisons was applied were appropriate. All error bars shown represent the standard error of the mean. Significance is indicated as follows: *: *p* ≤ 0.05 **: *p* ≤ 0.01; ***: *p* ≤ 0.001. 

## 4. Conclusions

Tetraspanins regulate a wide variety of cell-cell fusion processes, including syncytium formation induced by HIV-1 Env. We found that overexpression of tetraspanins blocks HIV-1-driven fusion after hemifusion but before pore expansion. To the best of our knowledge, this is the first description of a virus-associated host cell factor which regulates cell-cell fusion at a post-hemifusion stage. As such, this finding also provides a basis for further studies aimed at elucidating how tetraspanins can negatively regulate membrane fusion processes, both viral and non-viral.
